# A survey of the specificity and mechanism of 1,6 hexanediol-induced disruption of nuclear transport

**DOI:** 10.1080/19491034.2023.2240139

**Published:** 2023-07-27

**Authors:** Elizabeth C. Riquelme Barrientos, Tegan A. Otto, Sara N. Mouton, Anton Steen, Liesbeth M. Veenhoff

**Affiliations:** European Research Institute for the Biology of Ageing, University of Groningen, University Medical Center Groningen, 9713 AV Groningen, Groningen, The Netherlands

**Keywords:** 1,6-hexanediol, aliphatic alcohol, baker’s yeast, Karyopherin, liquid-liquid phase separation, Nuclear pore complex, nuclear transport, Nuclear transport receptors

## Abstract

Selective transport through the nuclear pore complex (NPC) depends on the dynamic binding of FG-repeat containing nucleoporins, the FG-nups, with each other and with Karyopherins (Kaps). Here, we assessed the specificity and mechanism by which the aliphatic alcohol 1,6-hexanediol (1,6HD) disrupts the permeability barrier of NPCs in live baker’s yeast cells. After a 10-minute exposure to 5% 1,6HD, no notable changes were observed in cell growth, cytosolic pH and ATP levels, or the appearance of organelles. However, effects on the cytoskeleton and Hsp104 were noted. 1,6HD clearly affected the NPC permeability barrier, allowing passive nuclear entry of a 177kDa reporter protein that is normally confined to the cytosol. Moreover, multiple Kaps were displaced from NPCs, and the displacement of Kap122-GFP correlated with the observed passive permeability changes. 1,6HD thus temporarily permeates NPCs, and in line with Kap-centric models, the mechanism includes the release of numerous Kaps from the NPCs.

## Introduction

The Nuclear Pore Complex (NPC) is the sole gate between the nucleus and cytosol. It is a large macromolecular assembly composed of about 30 conserved nucleoporins (nups). The central channel of NPCs is lined with intrinsically disordered phenylalanine-glycine rich nups, the FG-nups, and it hosts many nuclear transport receptors (NTRs), many belonging to the family of Karyopherins (Kaps) [1–4]. The NTRs bind their cargo and shuttle them through the channel by transiently binding the FG-nups [4–6]. For the NTR Importinβ it was shown that besides a fraction that is shuttling cargo between the cytoplasm and nucleus, there is also a fraction that is more stably associated with NPCs [7,8]. In addition to NTRs, also cargo and non-cargo are present in the NPC. In isolated yeast NPCs, 15,6 MDa worth of NTRs and 10,4 MDa worth of cargo add significantly to the 52,3 MDa mass of the nucleoporins [9]. The central channel of the nuclear pore complex is thus a highly crowded and complex environment where the joint presence of NTRs, FG-nups and cargo creates an environment that allows fast and selective transport.

The exact structure of the central channel has remained elusive because experimentally probing its behavior in living cells is challenging. Our knowledge about the behavior of the FG-nups and NTRs is inferred from, amongst others, imaging detergent-perforated or live cells [10–13]), AFM measurements on nuclear envelopes (NEs) [14], transport measurement in biomimetic NPCs [15–17], surface anchored FG-nups [7] or from probing the structural conformation of purified FG-nups or FG-nup fragment preparations [18–22]. These experimental studies, together with computational strategies [23–27], have resulted in a number of models explaining the fast and selective transport through the NPC [1–4,28,29]. All models agree that the phenylalanines of the FG-repeat regions that are engaging in hydrophobic interactions, as well as the intrinsically disordered nature of the FG-nups, are key parameters. They enable the highly dynamic intra- and inter-chain hydrophobic interactions between FG-repeat regions and with the hydrophobic grooves on the surfaces of NTRs. In the Kap-centric models the slow exchanging pool of NTRs are proposed to be important to create the proper barrier function [30–32].

Early experiments using aliphatic alcohols pointed to the importance of hydrophobic interactions for import into nuclei of permeabilized cells [33] and in live yeast cells [34]. Early experiments in permeabilized HeLa cells showed that selective transport of fluorescent reporters (MBP or IBB-MBP) was abrogated in the presence of hexane-1,2-diol but not by the less hydrophobic hexane-1,2,3-triol [33]. In live yeast cells, it was observed that the nuclear accumulation of GFP fused to a classical nuclear localization signal (NLS) was lost upon addition of alcohols and the extent of equilibration was dependent on the hydrophobicity of the alcohol [34]. Biochemical studies using purified FG-repeat fragments show that some of them are cohesive and that their interactions are disrupted by 1,6HD [35,36]. Also, within the yeast cytosol such overexpressed fragments form foci that are dispersed by 1,6HD [35]. Lastly, 1,6 HD was shown to increase the diameter of NPCs in *Xenopus* oocyte NE preparations [37]. Most dramatically, in the context of mutant NPCs that lack the inner ring nucleoporins Nup170 or Nup188, 1,6HD can even lead to loss of FG-nups from these NPCs [34,38]. The effect of hexanediol in the above studies was attributed to a reversible disruption of inter-FG repeat cohesion. However, as also the interactions between NTRs and FG-nups are based on hydrophobic interactions, hexanediol will likely also take effect here. Illustrative for the high surface hydrophobicity of NTRs, is their strong binding to a phenyl sepharose chromatography column yielding highly enriched fractions from HeLa cell extracts [33]. Jointly, these studies support the importance of hydrophobic interaction for nuclear transport, and the potential of 1,6 HD to disrupt those.

Unrelated to nuclear transport, 1,6HD has also been widely used to dissolve liquid–liquid phase separated compartments in cells and to dissolve condensates in *in vitro* studies. With aggregation-prone peptides, the alcohol dissolves hydrogels [39–41] but not fibers [42,43]. In cells, the interpretation of the effects of 1,6HD are more difficult [39] and depending on the cell type, growth condition and the concentration and length of treatment different results may be obtained. There are many examples of discrepancies in the literature; only one example is the organization of actin and tubulin. While some reports show that they are affected by 1,6HD [39,44], others report that microtubules are unaffected [42].

From the above, the question arises how specific the effects of 1,6HD on nuclear transport are, and whether they are based on a loss of cohesion between the FG-repeat regions, or between FG-nups and NTRs, or both. Here, we probe the impact of 1,6HD on nuclear transport and on the cellular localization of Nups and NTRs. We also assess a large number of possible indirect effects of 1,6HD, namely cell viability, the pH and ATP levels in the cytosol, and the appearance of mitochondria, Golgi, peroxisomes, ER, vacuoles, plasma membrane, nucleolus, secretory pathway, stress granules, the cytoskeleton and Hsp104 foci. Our data support that 1,6HD provides an intervention to temporarily increase the passive permeability of NPCs, and we show that the release of NTRs from the NPC is part of the mechanism.

## Results

### Disruption of the permeability barrier of NPCs by 1,6 hexanediol

To characterize nuclear transport, the terms influx and efflux are used to describe the process of nuclear entry or exit which occurs by diffusion down the concentration gradient. For reporters lacking NLS or NES sequences like GFP, the rates of influx and efflux are identical [45,46] and simply reflect the passive permeability of the NPC. Import and export are used for NTR-dependent transport, which can result in nuclear accumulation or depletion, respectively.

**Figure 1.**
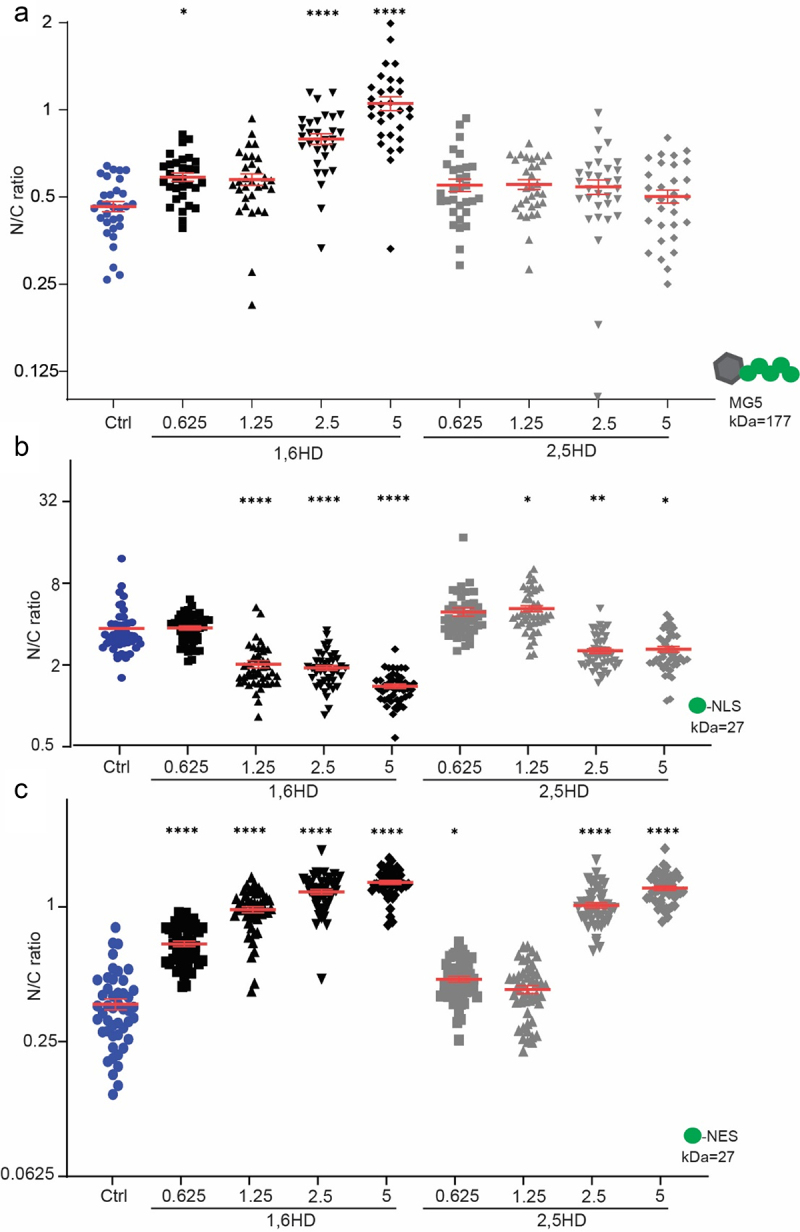
Disruption of NPC permeability barrier by 1,6HD. (a-c) Nuclear compartmentalization of GFP-based reporter proteins (MG5, GFP-NES, GFP-NLS) in yeast cells exposed for 10 min with the indicated concentrations of 1,6HD or 2,5HD. MG5 is a fusion of Maltose Binding Protein and 5 GFPs; GFP-NLS features the classical Simian Virus 40 NLS and GFP-NES the Stress-Seventy subfamily B1 NES. The N/C ratio is the ratio of the average fluorescence in the nucleus (N) over that in the cytoplasm (c). One-way ANOVA with Dunnett’s multiple comparison test comparing treatment to control was used to calculate the statistical significance in panel a and C and the non-parametrical Kruskal-Wallis with Dunn’s multiple comparison test in B. Error bars reflect the Standard Error of the Mean (SEM) of three independent experiments and a total of at least 30 cells per condition. P-values*<0,05 **<0,01 ****<0,0001.

Previous reports already showed that 1,6HD impacts import in yeast cells (Shulga and Goldfarb 2003 [35]. We add to this work and provide a quantitative analysis of the effects of 1,6HD and 2,5 HD on the influx of a large reporter consisting of a Maltose Binding Protein with 5 GFPs (MG5), and possessing a molecular weight of 177 kDa. The influx of such a large reporter is slow and in wild type cells this reporter is excluded from the nucleus [26]. Mid-exponential growing cells were exposed for 10 min to either 1,6HD or to the less hydrophobic alcohol 2,5-hexanediol (2,5HD), and the steady-state distribution of MG5 was determined as the ratio of the mean fluorescence in the nucleus over the cytosol (N/C ratio) (Figure 1a). The treatment with 1,6HD resulted in an increase in the N/C ratio which reflects an increased influx of MG5. The effect of 1,6HD was concentration dependent and increased in the range between 0,625% and 5% 1,6HD. The effect of the less hydrophobic 2,5 HD on the localization of MG5 remained insignificant in this range. The increase of the influx of this large reporter upon exposure to 1,6HD implies that the NPCs have a more permeable barrier after exposure to 1,6HD.

We next assessed the effect of 1,6HD on the localization of GFP-NLS, which is imported by Kap60 and Kap95, and of GFP-NES, which is exported by Crm1. The efflux of GFP-NLS is fast due to its small size and hence the nuclear accumulation of GFP-NLS reflects the balance between continuous Kap60/Kap95-facilitated import and its efflux; a pump-leak cycle [46]. Similarly, the balance of CRM1-facilitated export of GFP-NES and its influx leads to a steady-state nuclear exclusion [46]. Changes in influx and efflux therefore readily change the N/C ratio of these small reporters and based on the concentration dependent 1,6HD-induced influx of MG5 (Figure 1a) one would expect a decrease in the accumulation of GFP-NLS and exclusion of GFP-NES. Indeed, assessing the impact of exposure to different concentrations of 1,6 HD, we find that the reporters for import and export showed a decline in their nuclear accumulation and exclusion, respectively (Figure 1b,c). Reporters imported by Kap104 (Nab2 NLS) and by Pse1/Kap121 (Pho4 NLS) also lose their nuclear accumulation when exposed to 1,6HD (Supplementary Figure S1). An increase in efflux and influx is the simplest interpretation of the data in Figure 1b c. However, as the N/C ratios only provide a measure of the ratio between two parameters, we cannot exclude additional changes in the rates of import and export.

We note that the localization of GFP-NLS and GFP-NES is more sensitive to 1,6HD and 2,5 HD than the localization of MG5. E.g. the localization of MG5 was insensitive to the presence of 5% 2,5HD but the N/C ratios of GFP-NLS and GFP-NES do already change at concentrations above 2,5%. While other explanations may apply, this difference could be explained by the reporter size dependency of influx and efflux [26,47] which predicts that a small increase in passive permeability may affect the influx and efflux of GFP but leave the influx of MG5 unaltered.

Regardless of the precise changes in the kinetics of import and export, we can conclude that exposure of live yeast cells to 1,6HD (10 min, 0.625–5%) leads to loss of nuclear compartmentalization and that this is, at least in part, a consequence of the increased passive permeability of NPCs as measured by the increased influx of MG5 (Figure 1a).

### On the specificity of 1,6HD toward disrupting nuclear transport

**Figure 2.**
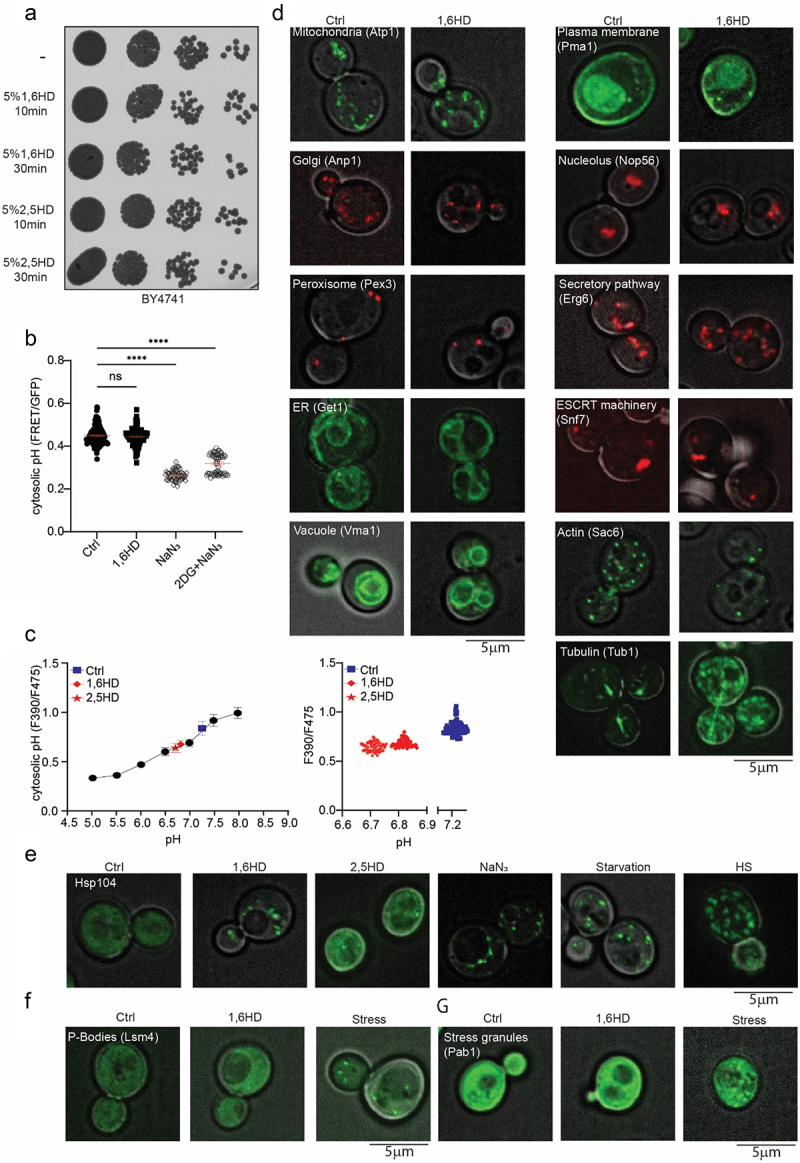
Impact of 1,6HD on growth, physiology and subcellular structures. (a) Growth assay showing serial dilutions of cultures exposed to 5% 1,6HD or 2,5HD for the indicated times. (b) Free ATP levels in cells measured using a FRET-based ATP-sensor; lower FRET/GFP ratio indicates lower free ATP. Cells were untreated (ctrl), exposed to 5% 1,6HD for 10 min, or exposed for 30 min to metabolic poisons azide (NaN_3_) or to NaN_3_ plus deoxyglucose (NaN_3_ +2DG). The error bar of the scatter plot reflects the SEM of three independent experiments. At least 60 cells per condition were analyzed. One-way ANOVA with Dunnett’s multiple comparison test was used to calculate the statistical significance of the difference in FRET/GFP ratios when comparing treatment to control. (c) Calibration curve for cytosolic pH values of the pH sensor pHluorin (F390/F475) in cells (black circles). The pH before (ctrl, blue squares) and after 10 min exposure to 1,6HD (red diamonds) or 2,5 HD (red stars) are indicated. Each point represents the mean and SEM from 60 cells (left graph), individual measurements are shown (right graph). (d) Fluorescence images of different cellular structures endogenously tagged with either GFP or mCherry, before and after 10 min exposure to 5% 1,6HD. (e) Fluorescence images showing localization of endogenously tagged Hsp104-GFP after 10 min exposure to 5% 1,6HD or 5% 2,5HD and under indicated stress conditions. (f,g) Fluorescence images showing localization of endogenously tagged Lsm4 (P-bodies, F) or Pab1 (Stress granules, G) with GFP after 10 min exposure with 5% 1,6HD and after induction of stress. Representative images of three independent replicates. The scales bars are 5 μm.

The question if the increased NPC permeability after exposure to 1,6HD is a direct consequence of an altered nuclear transport system, or rather a consequence of indirect effects on the cell’s physiology, is pertinent. Indeed, depending on the exposure time and concentration 1,6HD may well have pleotropic effects in cells, as also previously discussed [39]. Using the set concentration of 5% 1,6HD, we assessed all aspects of cell physiology that we deemed relevant and could assess. First, we treat the cells for 10 or 30 min with 5% 1,6HD or 2,5HD and observed no effects on growth (Figure 2a). Then, we assessed if 10 min exposure to 5% 1,6HD leads to changes in free ATP levels or cytosolic pH, using fluorescence-based sensors [48,49]. Our rationale for testing these was that ATP and pH levels could change when cells are experiencing metabolic stresses. We find, however, that the levels of free ATP are unchanged after 1,6HD treatment. As a control, sodium azide (NaN_3_) and 2-deoxy-glucose (2DG) were used, which both depleted the cell of energy (Figure 2b). The cytosolic pH values, calibrated as described in [50], decrease mildly from 7.2 to 6.8 or 6.7 after exposure to 1,6HD and 2,5HD, respectively, and therefore remain in the physiological range (Figure 2c).

Next, we looked at the morphology and localization of different subcellular structures using GFP- or RFP-tagged proteins marking the mitochondria, Golgi, peroxisome, ER, vacuole, plasma membrane, nucleolus, secretory pathway, and ESCRT machinery. From visual inspection, we conclude there are no obvious changes in their appearance after 10 min exposure to 5% 1,6HD (Figure 2d). In contrast, the appearance of microtubules and actin filaments does change after treatment with 1,6HD, which aligns with some previous literature [39,44]. Hsp104, a disaggregase that can refold and reactivate previously aggregated proteins and respond to alcohol-stress [51–54], forms foci upon exposure to 1,6HD, similar to when cells are exposed to either nitrogen starvation, energy depletion or heat shock (Figure 2e), suggesting that 1,6HD induces some level of protein stress. Finally, 1,6HD does not induce the formation of p-bodies (Figure 2f) or stress granules (Figure 2g).

Taking the above together, under the conditions where mid exponentially growing cells are exposed to 5% 1,6HD for 10 min, there are effects on the cytoskeleton and Hsp104 to be noted, but cell viability, the pH and ATP levels in the cytosol, and the appearance of mitochondria, Golgi, peroxisomes, ER, vacuoles, plasma membrane, nucleolus, the secretory and ESCRT pathways and stress granules are not notably changed. While this is not a proof of the absence of indirect effects on nuclear transport, the data suggest that the 1,6HD-dependent effects on NPC permeability shown in Figure 1 is due to direct effects on the nuclear transport machinery.

### 1,6HD induces the loss of NTRs from the NPCs in a manner that correlates with the disruption of the permeability barrier

Previous work proposed that the effects of 1,6HD are related to the alcohol-sensitive hydrophobic interactions between the FG-nups that maintain the permeability barrier [33,35,36]. Indeed, when the FG-domains of Nup100 (Nup100FG) in preformed condensates are exposed to the concentrations of 1,6HD that were also used in life cells (0–5%), partial solubilization of the condensates is observed (Supplementary Figure S2). While it is indeed expected that also *in vivo* 1,6 HD will alter the dynamic behavior of nups, additional explanations for the increased permeability of NPCs in 1,6HD treated cells relate to the composition of the NPCs and to the NTRs. We explore them both.

**Figure 3.**
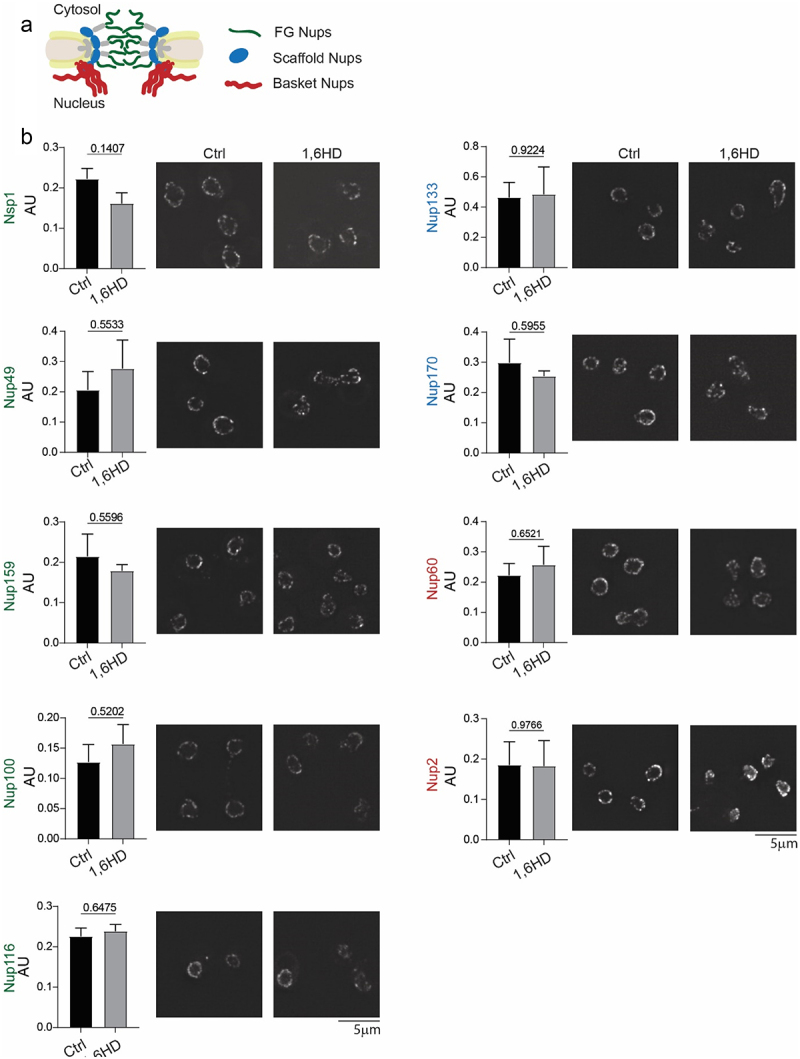
Impact of 1,6HD on the abundance and NE-localization of nups. (a) Cartoon representation of NPC indicating the position of the nups analyzed in B. (b) Endogenous Nup-GFP protein levels in whole cells lysates as determined by western blot before and after 10 min exposure to 5% 1,6HD; quantification gives mean, SEM and *P* values from unpaired t-test from at least three independent replicates. Fluorescence images of endogenously GFP-tagged nups before and after 10 min exposure with 5% 1,6HD. Representative images of three independent replicates. Shown are maximum projections of the 4 z-stacks around the z-stack that most clearly showed the NE. Supplementary Figure S2 shows maximum projections of the whole cell. The scale bar is 5 μm.

Previous work [34] showed that 1,6HD did not lead to a release of NPC components in wild type W303 cells but it did so in certain mutant backgrounds. We assessed the appearance of the NE and the abundance and localization of nups after exposure to 5% 1,6HD using nine representative endogenously tagged nups: five FG-nups (Nsp1, Nup49, Nup159, Nup100, Nup116), two scaffold nups (Nup133 and Nup170) and two basket nups (Nup60 and Nup2) in the here used strain background BY4741. A qualitative analysis of the images indicated that the morphology of the NE and the localization of the nups was similar in the presence and absence of 1,6 HD (Figure 3b and Supplementary Figure S3), consistent with [34], although more subtle effects may have remained undetected in this analysis. The fluorescent images could not be used to assess the expression levels of the nups as the fluorescence of GFP decreases in the presence of 1,6HD decreases (Supplementary Figure S4). Instead, proteins levels were analyzed by western blot, and this did not show significant changes (Figure 3b). We conclude that the 10 min 1,6HD treatment did not lead to major changes to the appearance of the NE or the localization and cellular abundance of the nups, suggesting that it does not lead to significant dissociation, aggregation, or degradation of the tested NPC components. Hence, we conclude that it is unlikely that the increased permeability is a result of changes to the nup-composition of the NPCs.

NPCs constitute a significant amount of NTRs at any point in time and their presence critically shapes the permeability barrier [8,9,16,55]. Therefore, we addressed the localization and abundance of endogenously GFP-tagged NTRs after treatment with 1,6HD. The interaction between the FG-nups and NTRs are based on dynamic multivalent binding with the phenylalanine’s of the FG-nups [4,21,22,28,56,57] and will thus also be sensitive to interventions disrupting hydrophobic interaction.

**Figure 4.**
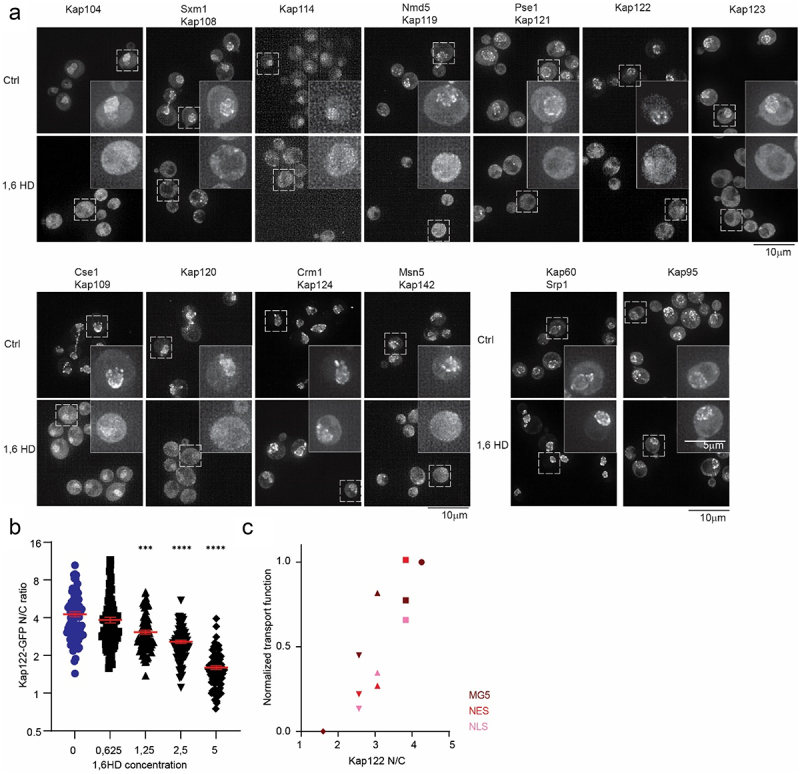
Impact of 1,6HD on NTRs. (a) Fluorescence images of endogenously GFP-tagged NTRs after 10 min exposure with 5% 1,6HD. Representative images of three independent replicates. The scale bar is 10 μm. Box indicates the zoomed-in single cell. (b) Nuclear accumulation of Kap122-GFP in yeast cells exposed for 10 min with the indicated concentrations of 1,6HD. Mean and SEM of three independent experiments; 90 cells per condition were analyzed; P-values from One-way ANOVA with Dunnett’s multiple comparison test ***<0,0005 ****<0,0001. (c) Average transport function measured with MG5 (dark red), GFP-NLS (pink) and GFP-NES (red) (from Fig1ABC but normalized on a scale from 0 to 1), as a function of Kap122-GFP location at the NE and nucleus (from Fig 4b) under control conditions and increasing concentrations of 1,6HD. Symbols as in 4B: 0% circles; 0,625% squares; 1,25% triangles up; 2,5% triangles down; 5% diamonds.

We evaluated the localization of endogenously GFP-tagged NTRs. Under normal conditions most NTRs are enriched at the NE showing a punctate rim staining, e.g., Kap109, and few are enriched in the nucleus, e.g. Kap104 (Figure 4a). Strikingly, the exposure to 1,6HD led to a clear relocalisation of NTRs (Figure 4a). Kap104, S×m1(Kap108), Kap114, Nmd5 (Kap119), Pse1 (Kap121), Kap122 and Kap123 lose their accumulation at the NE or nucleus upon exposure to 1,6HD and distribute over the cytosol and nucleus (Figure 4a). Cse1 (Kap109), Kap120, Crm1 (Kap124) and Msn5 (Kap142) which are normally enriched at the NE, partly relocate. Kap60 and Kap95 were not visibly affected by the treatment probably related to the previously described immobile pool of Kap95 at NPCs [8]. A higher concentration of 1,6HD or a longer exposure does alter the localizations of Kap95, Kap60 and Crm1 (Sup fig S5). When the less hydrophobic alcohol 2,5HD was used, it led to some NTRs losing their accumulation at the NE or nucleus, but always to a lesser extent compared to 1,6HD (Sup Fig S6). The alleviated effect of 2,5HD compared to 1,6HD on the localization of NTRs matches the generally milder effects on passive permeability (Figure 1a). We conclude that 1,6HD induces a reduction of the pool of NTRs at the NE and an increase in the nuclear or cytosolic pools, which we interpret as a release of NTRs from the NPCs.

In-line with Kap-centered models [55] and other reports showing the importantce of the NPC resident pool of NTRs for the passive selectivity of the permeability barrier [8,9,16,55], the massive relocation of NTRs from NPCs may mechanistically explain the 1,6HD-induced increase in the passive permeability of NPCs. To challenge this interpretation, we sought to quantitatively correlate the concentration-dependent NTR relocalisation, with the 1,6HD concentration-dependent entry of the reporters MG5 (Figure 1a), GFP-NLS (Figure 1b) and GFP-NES (Figure 1c). We chose Kap122 for this analysis as Kap122 clearly loses its accumulation at the NE and distributes over the cytosol (Figure 4a). The localization of endogenously tagged Kap122-GFP in the nucleus and NE was assessed in a strain co-expressing endogenously tagged Nup133-mCherry to mark the NE. The average nuclear accumulation of Kap122 gradually decreased from 4,3 to 3,8 to 3,1 to 2,6 to 1,6 upon exposure to zero, 0.625, 1.25, 2.5, or 5% 1,6HD. Moreover, we could correlate Kap122 relocalisation from the NE under these conditions with the measured passive permeability of NPCs for MG5, GFP-NLS, and GFP-NES with a Pearson correlation coefficient of 0.9, 0.8, and 0.9, respectively (Figure 4c). These correlations support that 1,6HD perturbs the NPC permeability barrier by releasing the NTRs.

## Discussion

Here we assessed the specificity and mechanism by which 1,6-hexanediol (1,6HD), an aliphatic alcohol that interferes with hydrophobic interactions, disrupts the permeability barrier of NPCs in live baker’s yeast cells. Exposure of live yeast cells to 1,6HD (10 min, 0–5%) leads to an increased passive permeability of NPCs. We conclude that this is likely a direct effect on the nuclear transport machinery as cell viability, the pH and ATP levels in the cytosol, and the appearance of mitochondria, Golgi, peroxisomes, ER, vacuoles, plasma membrane, nucleolus, secretory pathway and stress granules were not notably changed. There were effects on the cytoskeleton and protein homeostasis (Hsp104 foci) to be noted and we cannot exclude that 1,6 HD impacts the cell’s physiology in ways that we did not monitor. Mechanistically, we propose that the displacement of NTRs from the NPC underlies the loss of NPC function because 1,6HD treatment induced a massive relocation of multiple NTRs from NPCs. This displacement from the NE, as assessed for Kap122-GFP, quantitatively correlated with the increased passive permeability of NPCs.

Our studies align well with previous reports that showed that the selective properties of the FG-nups rely on the physical presence of NTRs within the NPC. The earliest study is one showing that the presence of transport factor enhances the selectivity of FG-nucleoporin-coated membranes [16]. The most recent reports on detergent-permeabilized human cells show that the enrichment of NTRs at the NPCs is important to keep the passive permeability low [31]. Our work adds to this by showing the importance of NTRs in live cells. The benefit being that in live cells there is a constant and large transport flux and therefore, together with the loss of the estimated 15,6 MDa of NTRs from the central channel also 10,4 MDa worth of cargo is being lost [9]. This joint loss of NTRs *and* cargo from the NPC central channel will present a major change in the macromolecular crowding and composition, and hence its physicochemical properties. How this alters the structural dynamics of the FG-nups, and if this poses a risk for NPC function would be interesting questions for the future.

Extrapolating from studies using purified FG-nup fragments that proposed that the effects of 1,6HD is related to the alcohol-sensitive hydrophobic interactions between the FG-nups [33,35,36] we expect that 1,6HD also alters the interactions between the FG-nups in our assays using live cells. This is, however, difficult to address in live cells. Hence, it remains unclear if the NTRs are released from the NPCs as a consequence of a lowered binding affinity between FG-nups, or because 1,6HD directly lowered the binding affinity of NTRs for the FG-repeat regions. If one considers that the functional composition of central channel is a system composed of NTRs *and* FG-nups in close collaboration, then the discrimination between these scenarios becomes less important.

An unanswered question in the field is if NPCs that become dysfunctional in time can be detected and removed. To assess this question, one needs to be able to inducibly damage NPCs. NPC permeabilization is expected to be an intervention that triggers quality control similar to when assembly fails [58–60]. The here described method could provide a tool to study the recruitment of quality control factors and to follow the repair or degradation.

Lastly, our study may serve as a warning that the effects of 1,6HD on liquid–liquid phase separation of diverse cellular macromolecular complexes may be a consequence of 1,6HD’s prime effect on the NPC and cognate NTRs. We speculate that the hydrophobic and highly acidic nature of NTRs may readily compromise their stability above a critical concentration. Consistent with this is that the overexpression of Sxm1, Kap95, and Kap114 is toxic to cells [61]. In any case, a major misplacement of NTRs and associated cargo will dramatically change the nuclear and cytoplasmic proteomes and this may generally compromise their stability. The increase in the number of Hsp104 foci that we observe may indeed reflect such loss of protein homeostasis.

Altogether, this paper provides a survey of the effects of 1,6HD on live cells and puts hydrophobic interactions between NTRs and FG-Nups center stage in the explanation how 1,6HD impacts NPC function.

## Materials and methods

### Strains and Growth conditions

All *Saccharomyces cerevisiae* strains used in this study have the BY4741 background, except yER016, which were created in the W303 background. Strains are listed in Table 1 and their genotype is described in Table 2. yER016, yER020, and yER023 were created as described in [64]. GFP-tagged strains were taken from the 4000-GFP yeast library (Thermofisher), RFP-tagged strains were taken from the localization database collection [63]. Cells were grown at 30°C, with shaking at 200 RPM on Synthetic Complete (SD) medium supplemented with 2% (w/v) glucose. Overnight cultures were diluted 10-fold in the morning and again for a second overnight culture. On the day of the experiment, cultures were diluted and grown for several hours to reach an OD_600_ 0.6–0.8 before each experiment. The cells expressing the different GFP-based reporters (GFP-NLS, GFP-NES, MG5) were grown overnight in synthetic dropout medium supplemented with 2% glucose, diluted 10-fold in 2% raffinose the next morning, and then again for an overnight culture in 2% raffinose. On the day of the experiment, cultures were diluted and induced for 3 H with 0.1% galactose reaching an OD of 0.4–0.6. For the MG5 reporter the expression was switched off for 1 H in 1% glucose before imaging to stop the induction and to allow for degradation of aggregates of the reporters that occasionally form.

**Table 1.** Key resources table.

Reagent type (species) or resource	Designation	Source or reference	Identifiers	Additional information
Gene (*S. cerevisiae*)	See table 2			
strain, strain background (*S. cerevisiae*)	BY4741	Invitrogen		
strain, strain background (*S. cerevisiae*)	BY4742	Invitrogen		
strain, strain background (*S. cerevisiae*)	W303	Invitrogen		
Genetic reagent (*S. cerevisiae*)	See table 2			
Antibody	Monoclonal antibody mouse anti-GFP	Santa Cruz	sc-9996	(1:500)
Antibody	Mouse IgG kappa binding protein conjugated to HRP; m-IgGK-BP-HRP	Santa Cruz	sc -516,102	(1:10000)
Recombinant DNA reagent	See table 3			
Sequenced-based reagent	Nup60_F	This paper	PCR primers GTTGATGAAAATAAAGTTGAGGCTTTCAAGTCCCTATATACCTTTCGTACGCTGCAGGTCGAC	
Sequenced-based reagent	Nup60_R	This paper	PCR primers TTGGGCTATACGGTAATTATGTCACGGCTAAAATTTTCATTATCAATCGATGAATTCGAGCTCG	
Sequenced-based reagent	Nup133_F	This paper	PCR primers GAAAAAAACTATACCATCAACTATGAAACCAACACTGTAGAATACGGTGACGGTGCTGG	
Sequenced-based reagent	Nup133_R	This paper	PCR primers CAGTAAAGTTTATTATATATATGTAAAATTGTATTATAGATATTATCGATGAATTCGAGCTCG	
Sequenced-based reagent	Pab1_F	This paper	PCR primers GTCTTTCAAAAAGGAGCAAGAACAACAAACTGAGCAAGCTCGTACGCTGCAGGTCGAC	
Sequenced-based reagent	Pab1_R	This paper	PCR primers GTTTGTTGAGTAGGGAAGTAGGTGATTACATAGAGCATTAATCGATGAATTCGAGCTCG	
chemical compound, drug	Yeast extract	BD	291946	
chemical compound, drug	Complete supplement mixture complete	Formedium	DCS0019	
chemical compound, drug	D-Glucose anhydrous	Fisher Chemical™	10141520	
chemical compound, drug	D-Raffinose pentahydrate	Thermo Scientific	195675000	
chemical compound, drug	D-Galactose	Acros Organics	150610010	
chemical compound, drug	Phosphatase buffered saline	Sigma-Aldrich	P4417	
chemical compound, drug	Tris base	Fisher Scientific™	BP152–1	
chemical compound, drug	HEPES	Fisher Scientific™	BP310–500	
chemical compound, drug	Sodium dodecyl sulfate (SDS) solution, 20%	SERVA	20767.03	
chemical compound, drug	EDTA	Sigma-Aldrich	ED2P–500	
chemical compound, drug	Triton X-100	Acros Organics	215682500	
chemical compound, drug	2-mercaptoethanol	Sigma-Aldrich	M6250–100	
chemical compound, drug	Sodium chloride	Acros Organics	207790010	
chemical compound, drug	Tween20	MP Biomedicals	TWEEN201	
chemical compound, drug	Magnesium chloride hexahydrate	Sigma-Aldrich	M2393	
chemical compound, drug	Sodium acetate anhydrous	Fisher Chemical™	S2080/53	
chemical compound, drug	Magnesium acetate tetrahydrate	Fisher Scientific™	BP215	
chemical compound, drug	Glycerol	Sigma-Aldrich	G5516	
chemical compound, drug	Phenylmethanesulfonyl fluoride (PMSF)	Sigma-Aldrich	P7626	
chemical compound, drug	cOmplete ULTRA tablets, Mini EDTA-free	Roche	05892791001	
chemical compound, drug	Albumine bovine serum (BSA)	Acros Organics	268131000	
chemical compound, drug	Glass beads	BioSpec Products	11079105	
chemical compound, drug	PierceTM BCA Protein Assay Kit	Fisher Scientific™	23225	
chemical compound, drug	ECL Prime Western Blotting Detection Reagent	Amersham	RPN2232	
chemical compound, drug	GX Stain-Free™ FastCast™ Acrylamide Kit, 10%	BioRad	1610183	
chemical compound, drug	PVDF Transfer Membrane	Thermo Scientific	88518	
chemical compound, drug	Methanol Technical	VWR	20903.368	
chemical compound, drug	IPTG	Sigma-Aldrich	10724815001	
chemical compound, drug	Ni sepharose	Cytiva	17531802	
chemical compound, drug	Guanidine hydrochloride	Thermo Scientific	24110	
chemical compound, drug	Brilliant blue G	Sigma-Aldricht	G-250	
chemical compound, drug	1,6 hexandiol	Sigma-Aldricht	240117–50	
chemical compound, drug	2,5 hexandiol	Sigma-Aldricht	H11904–50	
chemical compound, drug	Sodium azide	Sigma-Aldricht	S2002–100	
chemical compound, drug	2-deoxy-d-glucose	Sigma-Aldricht	D8375–1	
software, algorithm	Fiji	[67]		
software, algorithm	Resolve3D SoftWoRx	Cytiva

**Table 2.** Yeast strains used in this publication.

Strain BY4741^1^	Genotype	Source
yPP008; GFP-tcNLS	Mata his3Δ1 leu2Δ0 met15Δ0 ura3Δ0 GFP-tcNLS(pGal1):His Nup49-mCh:URA	[62]
yPP011; GFP-NES	Mata his3Δ1 leu2Δ0 met15Δ0 ura3Δ0 GFP-NES(pGal1):His Nup49-mCh:URA	[62]
GFP collection^2^	Mata his3Δ1 leu2Δ0 met15Δ0 ura3Δ0 **XX**-GFP:HIS3M X 6	ThermoFisher
Nup116-GFPboundary	Mata his3Δ1 leu2Δ0 met15Δ0 ura3Δ0	[62]
yER016; Nup60-GFP^1)^	Mata leu2–3, 112 trp1–1 can1–100 ura3–1 ade 2–1 his3–11, 15 Nup60-GFP:KanMX4	This paper
yIS010; Nup2-GFP Nup49mCherry	Mata his3Δ1 leu2Δ0 met15Δ0 ura3Δ0 Nup2-GFP:His3MX6 Nup49-mCherry:URA	[62]
yER020; Pab1-GFP	Mata his3Δ1 leu2Δ0 met15Δ0 ura3Δ0 Pab1-GFP:HIS3M X 6	This paper
RFP localization database^3^	Matα his3Δ1 leu2Δ0 lys2Δ0 ura3Δ0 **YY**-RFP:KanMX6	[63]
SMY12	Mata his3Δ1 leu2Δ0 met15Δ0 ura3Δ0 pTEF1-pHluorin:His3M X 6	[50]
SMY16	Mata his3Δ1 leu2Δ0 met15Δ0 ura3Δ0 ATP sensor pTEF1-his6-ymEGFP Δ11-B.subtilis ε-ymScarletI:HIS3M X 6	[61]
yER023; Kap122-GFP Nup133mCherry	Mata his3Δ1 leu2Δ0 met15Δ0 ura3Δ0 Kap122-GFP:HIS3M X 6 Nup133-mCherry:URA	This paper
yAS49; Nup133-mCherry	Mata his3Δ1 leu2Δ0 met15Δ0 ura3Δ0 Nup133-mCherry:URA	This paper

^1^yER016 is in W303 background

^2^**XX** is: NSP1, Nup49, Nup100, Nup133, Nup159, Nup170, LSM4, Hsp104, ATP1, Get1, Vma1, Pma1, Tub1, Kap124, Kap95, Kap60, Kap122, Kap104, Kap142, Kap119, Kap121, Kap108, Kap109, Kap114, Kap120, Kap123.

^3^**YY** is: Anp1, Pex3, Nop56, Erg6, Snf7.

**Table 3.** Plasmids used in this publication.

Plasmid number	Genotype	Source
pPP008; MG5	pUG34-Gal1-MBP-5XGFP-His	[26]
pACM063; mCh-L-TM	pUG36-Gal-mCherry linker-TM-URA	[45]
pYM28	pAgTEF-SpHIS5-tAgTEF	Euroscarf [64],
pYM30	pAgTEF-kanMX-tAgTEF	Euroscarf [64],
pPP014	mCherry-Ura cassette	[62]
pRS303-NLSNab2-GFP	NLSNab2-GFP	[46]
pRS303-NLSPho4-GFP	NLSPho4-GFP	[46]

### Spot assay

10 mL of yeast culture was grown to an OD600 0.3–0.4 and treated with 5% 1,6HD or 5% 2,5HD for 10 or 30 min, as indicated in Figure 2a, and diluted in sterilized milliQ water to obtain 10^6^ cells/ml, and further serial diluted in milliQ water. 5 μl of each dilution was spotted on YPD plates and the plates were imaged after 48 H growth at 30°C.

### Microscopy

All *in vivo* experiments were performed at 30°C. Images were acquired using a DeltaVision Elite imaging system (Cytiva) composed of an inverted microscope (IX-71; Olympus) equipped with a UPlanSApo 100× (1.4 NA) oil immersion objective, InsightSSI solid-state illumination, and an EDGE sCMOS 5.5 camera. For all experiments, stacks of 30 images with 0.2 μm spacing were taken.

### Protein lysate and Western Blot

20 ml of yeast culture was grown to an OD_600_ 0.8–1.2. Cells were subsequently treated with 5% 1,6HD for 10 min at 30°C, with shaking at 200 RPM. After the treatment, cells were centrifuged, and all the following steps were performed at 4°C. The cells were resuspended in 0.25 ml of lysis buffer (50 mM HEPES, 200 mM sodium acetate, 1 mM EDTA, 5 mM magnesium acetate, 5% glycerol, 1% triton x-100, 10 mM β-mercaptoethanol, protease inhibitor without EDTA) and lysed in two rounds of bead-beating in a Fastprep device (MP Biomedicals). Lysates were cleared by consecutive centrifugation steps at 6000 × g for 5 min and twice at 17,700 × g for 5 min. Western blots were performed as follows: whole cell lysates were separated by SDS-PAGE. The proteins were subsequently transferred to PVDF membranes. After blocking with 5% skim milk in TBS-T, GFP-tagged proteins were detected with anti-GFP (Santa Cruz sc-9996 HRP), followed by HRP-conjugated mouse IgG kappa-binding protein (Santa Cruz sc -516,102, m-igGK BP-HRP).

### Expression and purification of nucleoporin FG-domains

Nup100FG domains were expressed and purified as described in [65]. In short: FG-domains proteins with an N terminal His-tag and a unique C-terminal cysteine were expressed in *Escherichia coli*, by induction with 0.5 mM IPTG and purified from cell extracts on a Nickel-Sepharose column under denaturing conditions (2 M GuHCl, 100 mM Tris-HCl pH 8). The C-terminal cysteine was reduced with DTT and blocked by modification with Iodoacetamide. Protein purity was checked with SDS-PAGE and subsequent Brilliant Blue staining.

### Spin Assay

A concentrated stock of 100 μM Nup100FG domains in 2 M GuHCl, 100 mM Tris-HCl pH 8, was diluted to 3 μM into TBS (50 mM Tris-HCl, 150 mM NaCl pH 8). The protein was left to self-assemble into particles for 1 h at RT, and then the protein was treated for 10 min with different concentrations of 1,6HD. Samples were centrifuged (17.700 × g for 10 min at RT), and soluble and insoluble fractions were run separately on SDS PAA gels. Gels were stained with Brilliant Blue G (Sigma-Aldrich, G-250) and imaged using a BioRad chemidoc (BioRad). Band intensities were determined using Fiji (Image J, National Institute of Health).

### Determining the intracellular pH with the pHluorin sensor

pHluorin ratios were calibrated in live cells in buffers with a pH of 5, 5.5, 6, 6.5, 7, 7.5, and 8, as described in [50]. The F390/F475 ratios were determined from cells on a glass slide. Cells were then treated with 1,6HD as described, and a calibration curve was used to determine the pH change after treatment.

### ATP sensor values and free ATP levels

Cells expressing a FRET-based ATP sensor [61], were used to determine free ATP levels as described in [61]. Cells were treated as described, imaged, and the FRET over GFP ratio was calculated using Fiji (see below).

### Image Analysis

All images were processed using Fiji (Image J, National Institute of Health). For each image, the z-stack with the best focus was selected. For the pHluorin and the ATP sensor, we determined the fluorescence in each channel corrected for the background, and determined the ratio between them. To quantify N/C ratios of the GFP-based reporters and Kap122, the average fluorescent intensity in the nucleus and the cytosol was measured. The nucleus was outlined using either the NE/ER marker mCherry-TM (pACM063) (Figure 1) or Nup133-mCherry (Figure 4b). A section of the cytosol excluding the vacuole was selected to measure the fluorescence in the cytosol.

### Statistical Analysis

Statistical parameters, including the number of cells analyzed, are reported in figure legends. All regressions and correlations leading to the sigmoidal curve equation, R^2^, and all Pearson’s correlation statistics were done in GraphPad Prism [43,66].

## Supplementary Material

Supplemental Material

## Reagent availability

All reagents are available upon request.

## References

[1] Dultz E, Wojtynek M, Medalia O, et al. The nuclear pore complex: birth, life, and death of a cellular behemoth. Cells. 2022;11(9):1–17. doi: 10.3390/cells11091456. PMC9100368 35563762

[2] Javier F-M, Rout MP. One ring to rule them all? Structural and functional diversity in the nuclear pore complex. Trends Biochem Sci. 2021;46(7):595–607. doi: 10.1016/j.tibs.2021.01.003 33563541 PMC8195821

[3] Hampoelz B, Andres-Pons A, Kastritis P, et al. Structure and assembly of the nuclear pore complex. Annu Rev Biophys. 2019;48:515–536. doi: 10.1146/annurev-biophys-052118-115308 30943044

[4] Wing CE, Yee Joyce Fung H, Min Chook Y. Karyopherin-mediated nucleocytoplasmic transport. Nat Rev Mol Cell Biol. 2022;23(5):307–328. doi: 10.1038/s41580-021-00446-7 35058649 PMC10101760

[5] Bayliss R, Littlewood T, Stewart M. Structural Basis for the Interaction between FxFG nucleoporin repeats and importin-β in nuclear trafficking. Cell. 2000;102(1):99–108. doi: 10.1016/S0092-8674(00)00014-3. 10929717

[6] Giulia P, Caria J, Lemke EA. Cargo Transport through the Nuclear Pore Complex at a Glance. J Cell Sci. 2021 2;134. doi: 10.1242/jcs.247874 33495357

[7] Kapinos LE, Schoch RL, Wagner RS, et al. Karyopherin-centric control of nuclear pores based on molecular occupancy and kinetic analysis of multivalent binding with FG Nucleoporins. Biophys J. 2014;106(8):1751–1762. doi: 10.1016/j.bpj.2014.02.021 24739174 PMC4008817

[8] Lowe AR, Tang JH, Yassif J, et al. Importin-β modulates the permeability of the nuclear pore complex in a ran-dependent manner. Elife. 2015;2015(4):1–24. doi: 10.7554/eLife.04052. PMC4375889 25748139

[9] Kim SJ, Fernandez-Martinez J, Nudelman I, et al. Integrative structure and functional anatomy of a nuclear pore complex. Nature. 2018;555(7697):475–482. doi: 10.1038/nature26003 29539637 PMC6022767

[10] Rajdeep C, Sau A, Musser SM. Super-resolved 3D tracking of cargo transport through nuclear pore complexes. Nat Cell Biol. 2022;24(1):112–122. doi: 10.1038/s41556-021-00815-6 35013558 PMC8820391

[11] Mattheyses AL, Kampmann M, Atkinson CE, et al. Fluorescence anisotropy reveals order and disorder of protein domains in the nuclear pore complex. Biophys J. 2010;99(6):1706–1717. doi: 10.1016/j.bpj.2010.06.075 20858414 PMC2941012

[12] Schnell SJ, Tingey M, Yang W. Speed microscopy: high-speed single molecule tracking and mapping of nucleocytoplasmic transport. Methods Mol Biol. 2022;2502:353–371. doi: 10.1007/978-1-0716-2337-4_23 35412250 PMC10131132

[13] Yu M, Heidari M, Mikhaleva S, et al. Visualizing the disordered nuclear transport machinery in situ. Nature. 2023;617(7959):162–169. 10.1038/s41586-023-05990-0 37100914 PMC10156602

[14] Yusuke S, Mazur A, Kapinos LE, et al. Spatiotemporal dynamics of the nuclear pore complex transport barrier resolved by high-speed atomic force microscopy. Nature Nanotechnol. 2016;11(8):719–723. doi: 10.1038/nnano.2016.62 27136131

[15] Fisher PDE, Shen Q, Akpinar B, et al. A programmable DNA origami platform for organizing intrinsically disordered nucleoporins within nanopore confinement. ACS Nano. 2018;12(2):1508–1518. doi: 10.1021/acsnano.7b08044 29350911 PMC5834394

[16] Tijana J-T, Tetenbaum-Novatt J, Sophia McKenney A, et al. Artificial nanopores that mimic the transport selectivity of the nuclear pore complex. Nature. 2009;457(7232):1023–1027. doi: 10.1038/nature07600 19098896 PMC2764719

[17] Kowalczyk SW, Kapinos L, Blosser TR, et al. Single-molecule transport across an individual biomimetic nuclear pore complex. Nature Nanotechnol. 2011;6(7):433–438. doi: 10.1038/nnano.2011.88 21685911

[18] Ader C, Frey S, Maas W, et al. Amyloid-like Interactions within Nucleoporin FG Hydrogels. Proc Natl Acad Sci USA. 2010;107(14):6281–6285. doi: 10.1073/pnas.0910163107 20304795 PMC2852002

[19] Giorgia C, Paci G, Caria J, et al. The liquid state of FG-Nucleoporins mimics permeability barrier properties of nuclear pore complexes. J Cell Bio. 2020 1;219. doi: 10.1083/jcb.201907157 PMC7039189 31723007

[20] Steffen F, Richter RP, Görlich D. FG-Rich Repeats of Nuclear Pore Proteins Form a Three-Dimensional Meshwork with Hydrogel-like Properties. Science. 2006;314(5800):815–817. doi: 10.1126/science.1132516 17082456

[21] Hayama R, Sparks S, Hecht LM, et al. Thermodynamic characterization of the multivalent interactions underlying rapid and selective translocation through the nuclear pore complex. J Biol Chem. 2018;293(12):4555–4563. doi: 10.1074/jbc.AC117.001649 29374059 PMC5868264

[22] Sparks S, Temel DB, Rout MP, et al. Deciphering thE ‘fuzzy’ interaction of FG nucleoporins and transport factors using small-angle neutron scattering. Structure. 2018;26(3):477–484.e4. doi: 10.1016/j.str.2018.01.010 29429880 PMC5929991

[23] Davis LK, Ford IJ, Hoogenboom BW. Crowding-induced phase separation of nuclear transport receptors in FG nucleoporin assemblies. Elife. 2022;11:1–20. doi: 10.7554/eLife.72627 PMC8880993 35098921

[24] Ghavami A, Veenhoff LM, Van Der Giessen E, et al. Probing The disordered domain of the nuclear pore complex through coarse-grained molecular dynamics simulations. Biophys J. 2014;107(6):1393–1402. doi: 10.1016/j.bpj.2014.07.060 25229147 PMC4167297

[25] Isgro TA, Schulten K. Association of Nuclear Pore FG-Repeat Domains to NTF2 Import and Export Complexes. J Mol Biol. 2007;366(1):330–345. doi: 10.1016/j.jmb.2006.11.048. 17161424

[26] Popken P, Ghavami A, Onck PR, et al. Size-dependent leak of soluble and membrane proteins through the yeast nuclear pore complex. ?Mol Biol Cell. 2015;26(7):1386–1394. doi: 10.1091/mbc.E14-07-1175 25631821 PMC4454183

[27] Zheng T, Zilman A. Self-regulation of the nuclear pore complex enables clogging-free crowded transport. PNAS. 2023;120(7). doi: 10.1073/pnas.2212874120 PMC9963888 36757893

[28] Hoogenboom BW, Hough LE, Lemke EA, et al. Physics of the nuclear pore complex: theory, modeling and experiment. Phys Rep. 2021;921:1–53. doi: 10.1016/j.physrep.2021.03.003 35892075 PMC9306291

[29] Huang K, Szleifer I. Modeling the nucleoporins that form the hairy pores. Biochem Soc Trans. 2020;48(4):1447–1461. doi: 10.1042/BST20190941. 32794558

[30] Fragasso A, de Vries HW, Andersson J, et al. Transport receptor occupancy in nuclear pore complex mimics. Nano Res. 2022;15(11):9689–9703. doi: 10.1007/s12274-022-4647-1

[31] Kalita J, Kapinos LE, Zheng T, et al. Karyopherin enrichment and compensation fortifies the nuclear pore complex against nucleocytoplasmic leakage. J Cell Bio. 2022 3;221. doi: 10.1083/jcb.202108107 PMC8932525 35089308

[32] Kapinos LE, Huang B, Rencurel C, et al. Karyopherins regulate nuclear pore complex barrier and transport function. J Cell Bio. 2017;216(11):3609–3624. doi: 10.1083/jcb.201702092 28864541 PMC5674887

[33] Ribbeck K, Görlich D. The permeability barrier of nuclear pore complexes appears to operate via hydrophobic exclusion. Embo J. 2002;21(11):2664–2671. doi: 10.1093/emboj/21.11.2664 12032079 PMC126029

[34] Nataliya S, Goldfarb DS. Binding dynamics of structural nucleoporins govern nuclear pore complex permeability and may mediate channel gating. Mol Cell Biol. 2003;23(2):534–542. doi: 10.1128/mcb.23.2.534-542.2003 12509452 PMC151542

[35] Patel SS, Belmont BJ, Sante JM, et al. Natively unfolded nucleoporins gate protein diffusion across the nuclear pore complex. Cell. 2007;129(1):83–96. doi: 10.1016/j.cell.2007.01.044 17418788

[36] Schmidt HBR, Görlich D. Nup98 FG domains from diverse species spontaneously phase-separate into particles with nuclear pore-like permselectivity. Elife. 2015;4:1–30. doi: 10.7554/eLife.04251 PMC4283134 25562883

[37] Jäggi RD, Franco-Obregón A, Mühlhäusser P, et al. Modulation of Nuclear Pore Topology by Transport Modifiers. Biophys J. 2003;84(1):665–670. doi: 10.1016/S0006-3495(03)74886-3 12524319 PMC1302647

[38] Onischenko E, Tang JH, Andersen KR, et al. Natively Unfolded FG repeats stabilize the structure of the nuclear pore complex. Cell. 2017;171(4):904–917.e19. doi: 10.1016/j.cell.2017.09.033 29033133 PMC5992322

[39] Kroschwald S, Maharana S, Simon A. Hexanediol: a chemical probe to investigate the material properties of membrane-less compartments. Matters. 2017;1–7. doi: 10.19185/matters.201702000010

[40] Molliex A, Temirov J, Lee J, et al. Phase separation by low complexity domains promotes stress granule assembly and drives pathological fibrillization. Cell. 2015;163(1):123–133. doi: 10.1016/j.cell.2015.09.015 26406374 PMC5149108

[41] Shi KY, Mori E, Nizami ZF, et al. Toxic PRn Poly-dipeptides encoded by the C9orf72 repeat expansion block nuclear import and export. Proc Natl Acad Sci USA. 2017;114(7):E1111–17. doi: 10.1073/pnas.1620293114 28069952 PMC5320981

[42] Lin Y, Mori E, Kato M, et al. Toxic PR Poly-Dipeptides Encoded by the C9orf72 repeat expansion target LC domain polymers. Cell. 2016;167(3):789–802.e12. doi: 10.1016/j.cell.2016.10.003 27768897 PMC5076566

[43] Van Lindt J, Lazar T, Pakravan D, et al. F/YGG-Motif is an intrinsically disordered nucleic-acid binding motif. RNA Biol. 2022;19(1):622–635. doi: 10.1080/15476286.2022.2066336. 35491929 PMC9067507

[44] Wheeler JR, Matheny T, Jain S, et al. Distinct stages in stress granule assembly and disassembly. Elife. 2016;5(Se):1–25. doi: 10.7554/eLife.18413. PMC5014549 27602576

[45] Meinema AC, Poolman B, Veenhoff LM. Quantitative analysis of membrane protein transport across the nuclear pore complex. Traffic. 2013;14(5):487–501. doi: 10.1111/tra.12048. 23357007

[46] Timney BL, Tetenbaum-Novatt J, Agate DS, et al. Simple kinetic relationships and nonspecific competition govern nuclear import rates in vivo. J Cell Bio. 2006;175(4):579–593. doi: 10.1083/jcb.200608141 17116750 PMC2064595

[47] Timney BL, Raveh B, Mironska R, et al. Simple rules for passive diffusion through the nuclear pore complex. J Cell Bio. 2016;215(1):57–76. doi: 10.1083/jcb.201601004 27697925 PMC5057280

[48] Hiromi I, Huynh Nhat KP, Togawa H, et al. Visualization of ATP levels inside single living cells with fluorescence resonance energy transfer-based genetically encoded indicators. Proc Nat Acad Sci. 2009;106(37):15651–15656. doi: 10.1073/pnas.0904764106 19720993 PMC2735558

[49] Miesenböck G, De Angelis DA, Rothman JE. Visualizing Secretion and Synaptic Transmission with PH-Sensitive Green Fluorescent Proteins. Nature. 1998;394(July):192–195. https://www.nature.com/articles/BF28190 9671304 10.1038/28190

[50] Mouton SN, Thaller DJ, Crane MM, et al. A Physicochemical Perspective of Aging from Single-Cell Analysis of Ph, Macromolecular and Organellar Crowding in Yeast. Elife. 2020;9:1–42. doi: 10.7554/ELIFE.54707 PMC7556870 32990592

[51] Bösl B, Grimminger V, Walter S. The Molecular Chaperone Hsp104-A Molecular Machine for Protein Disaggregation. J Struct Biol. 2006;156(1):139–148. doi: 10.1016/j.jsb.2006.02.004 16563798

[52] Glover JR, Lindquist S. Hsp104, Hsp70, and Hsp40: A Novel Chaperone System That Rescues Previously Aggregated Proteins. Cell. 1998;94(1):73–82. doi: 10.1016/S0092-8674(00)81223-4. 9674429

[53] Harari A, Zoltsman G, Levin T, et al. Hsp104 N-Terminal Domain Interaction with Substrates Plays a Regulatory Role in Protein Disaggregation. FEBS J. 2022;289(17):5359–5377. doi: 10.1111/febs.16441 35305079 PMC9541529

[54] Yolanda S, Lindquist SL. HSP104 Required for Induced Thermotolerance. Science. 1990;248(4959):1112–1115. doi: 10.1126/science.2188365 2188365

[55] Joanna K, Kapinos LE, Lim RYH. On the Asymmetric Partitioning of Nucleocytoplasmic Transport – Recent Insights and Open Questions. J Cell Sci. 2021;134(7). doi: 10.1242/jcs.240382 33912945

[56] Hough LE, Dutta K, Sparks S, et al. The Molecular Mechanism of Nuclear Transport Revealed by Atomic-Scale Measurements. Elife. 2015;4(September):1–23. doi: 10.7554/eLife.10027. PMC4621360 26371551

[57] Milles S, Mercadante D, Valle Aramburu I, et al. Plasticity of an Ultrafast Interaction between Nucleoporins and Nuclear Transport Receptors. Cell. 2015;163(3):734–745. doi: 10.1016/j.cell.2015.09.047 26456112 PMC4622936

[58] Thaller DJ, Tong D, Marklew CJ, et al. Direct Binding of ESCRT Protein Chm7 to phosphatidic acid-rich membranes at nuclear envelope herniations. J Cell Bio. 2021 3;220. doi: 10.1083/JCB.202004222 PMC7816628 33464310

[59] Thaller DJ, Allegretti M, Borah S, et al. An escrt-lem protein surveillance system is poised to directly monitor the nuclear envelope and nuclear transport system. Elife. 2019;8:1–36. doi: 10.7554/eLife.45284 PMC6461442 30942170

[60] Webster BM, Thaller DJ, Jäger J, et al. Chm7 and Heh1 collaborate to link nuclear pore complex quality control with nuclear envelope sealing. Embo J. 2016;35(22):2447–2467. doi: 10.15252/embj.201694574 27733427 PMC5109239

[61] Semmelink MFW, Jafarinia H, Wolters JC, et al. 2022. “Nuclear Transport under Stress Phenocopies Transport Defects in Models of C9Orf72 ALS,” 1–38. bioRxiv 2022.04.13.488135; doi: 10.1101/2022.04.13.488135

[62] Rempel IL, Crane MM, Thaller DJ, et al. Age-dependent deterioration of nuclear pore assembly in mitotic cells decreases transport dynamics. Elife. 2019;8(June):1–26. doi: 10.7554/eLife.48186 PMC6579512 31157618

[63] Huh WK, Falvo JV, Gerke LC, et al. Global analysis of protein localization in budding yeast. Nature. 2003;425(6959):686–691. http://yeastgfp.ucsf.edu. 14562095 10.1038/nature02026

[64] Janke C, Magiera MM, Rathfelder N, et al. A Versatile Toolbox for PCR-Based tagging of yeast genes: new fluorescent proteins, more markers and promoter substitution cassettes. Yeast. 2004;21(11):947–962. doi: 10.1002/yea.1142 15334558

[65] Kuiper EFE, Gallardo P, Bergsma T, et al. The chaperone DNAJB6 Surveils FG-Nucleoporins and is required for interphase nuclear pore complex biogenesis. Nat Cell Biol. 2022;24(11):1584–1594. doi: 10.1038/s41556-022-01010-x 36302971

[66] Springhower CE, Rosen MK, Min Chook Y. Karyopherins and Condensates. Curr Opinion Cell Biol. 2020;64:112–123. doi: 10.1016/j.ceb.2020.04.003 32474299 PMC7371519

[67] Schindelin J, Arganda-Carreras I, Frise E, et al. Fiji: an open-source platform for biological-image analysis. Nature Methods. 2012;9(7):676–682. doi: 10.1038/nmeth.2019 22743772 PMC3855844

