# Supplementary material for: A survey of the specificity and mechanism of 1,6 hexanediol-induced disruption of nuclear transport
Source: Nucleus. 2023 Jul 27;14(1):2240139. doi: 10.1080/19491034.2023.2240139 (PMC10376917; doi:10.1080/19491034.2023.2240139)
Supplement: Supplemental Material [file KNCL_A_2240139_SM4430.docx]

**
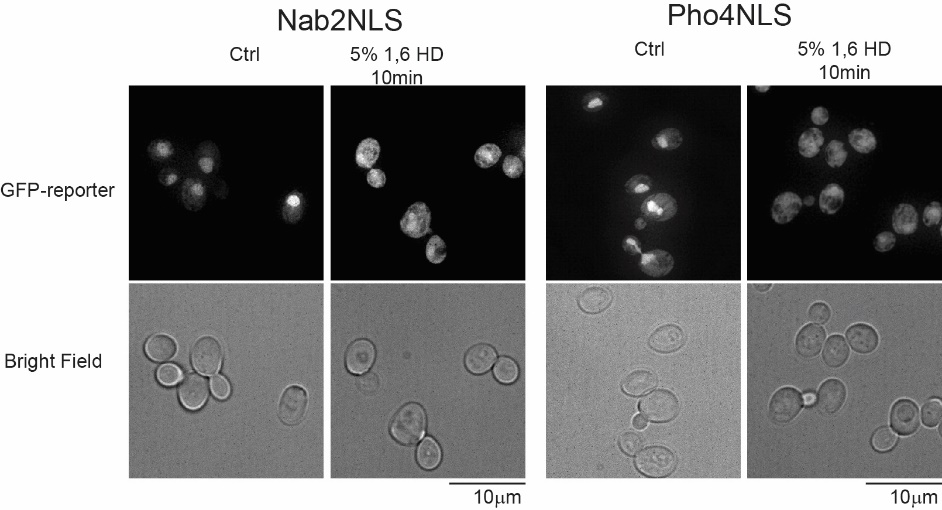
**

**Supplementary Figure 1.** Fluorescence images showing the loss of nuclear accumulation of GFP-Nab2NLS and GFP-Pho4NLS after exposure to 5% 1,6HD for 10 min.


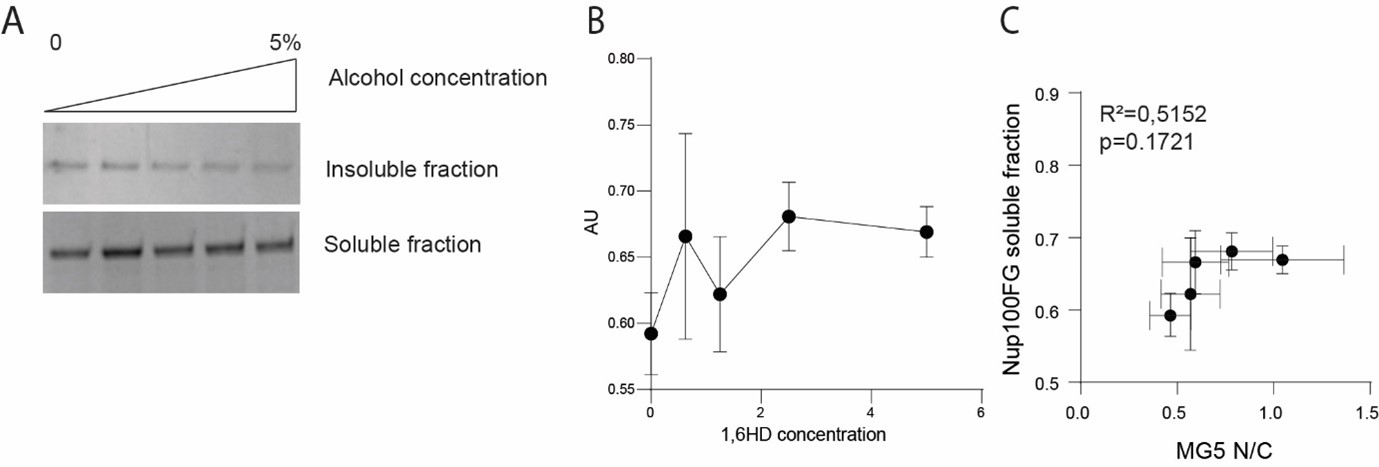


**Supplementary Figure 2.** (A) Purified Nup100FG domains were left to form condensates for 1 hour and subsequently treated for 10min with 0, 0.625, 1.25, 2.5 or 5% 1,6HD. Soluble and insoluble fractions were obtained by centrifugation, separated by SDS-PAGE and visualized by Brilliant Blue staining. Representative image of three independent experiments. (B) Quantification of the soluble fractions in (A) Error bars reflect SEM of three independent experiments. (C) Pearson correlation coefficient and two-tailed P values were calculated for the N/C ratio of reporter MG5 against the soluble fraction of Nup100FG domain after different concentrations of 1,6HD. Error bars reflect SEM from the mean of three independent experiments.


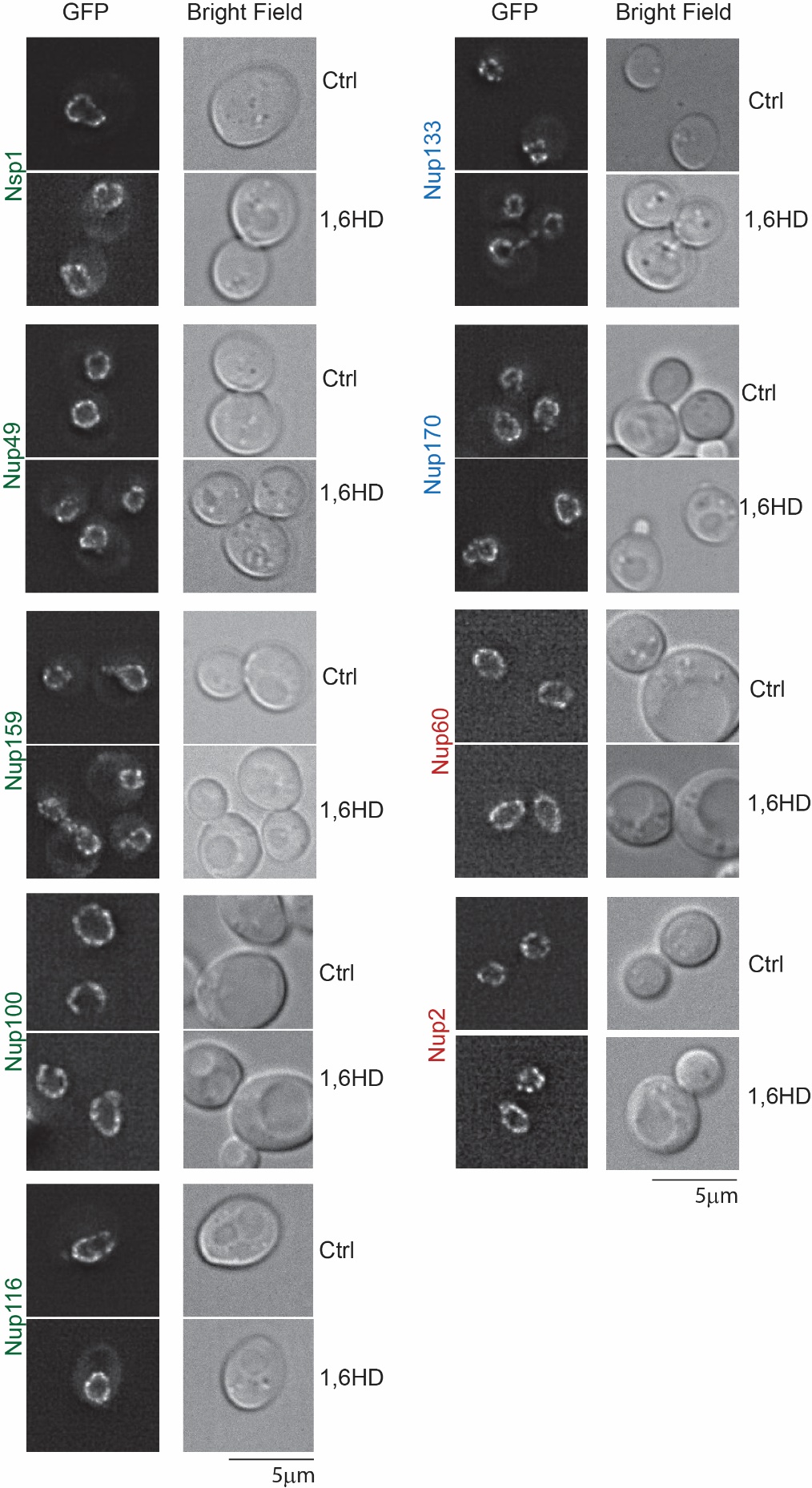


**Supplementary Figure 3. Impact of 1,6HD on the localization of nups.** Fluorescence images of endogenously GFP-tagged nups before and after 10 min exposure with 5% 1,6HD. Representative images of three independent replicates. Shown are maximum projections of the whole cell. The scale bar is 5μm.


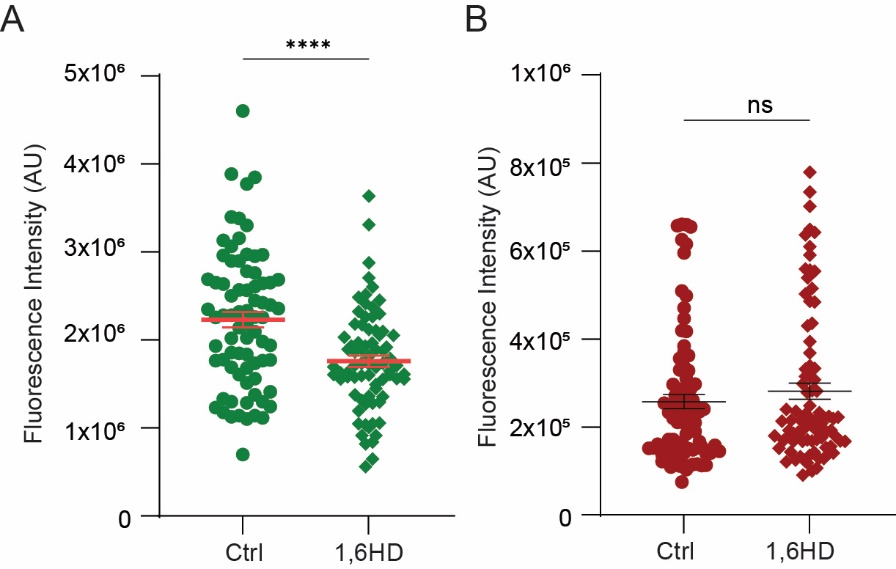


**Supplementary Figure 4.** Fluorescence intensity of endogenously tagged Nup133-GFP (A) and Nup133-mCherry (B) before and after 10 min of 5% 1,6HD. Mean and SEM of three independent experiments; at least 70 cells per condition were analysed; P-values from One-way ANOVA with Dunnett’s multiple comparison test ****<0,0001.


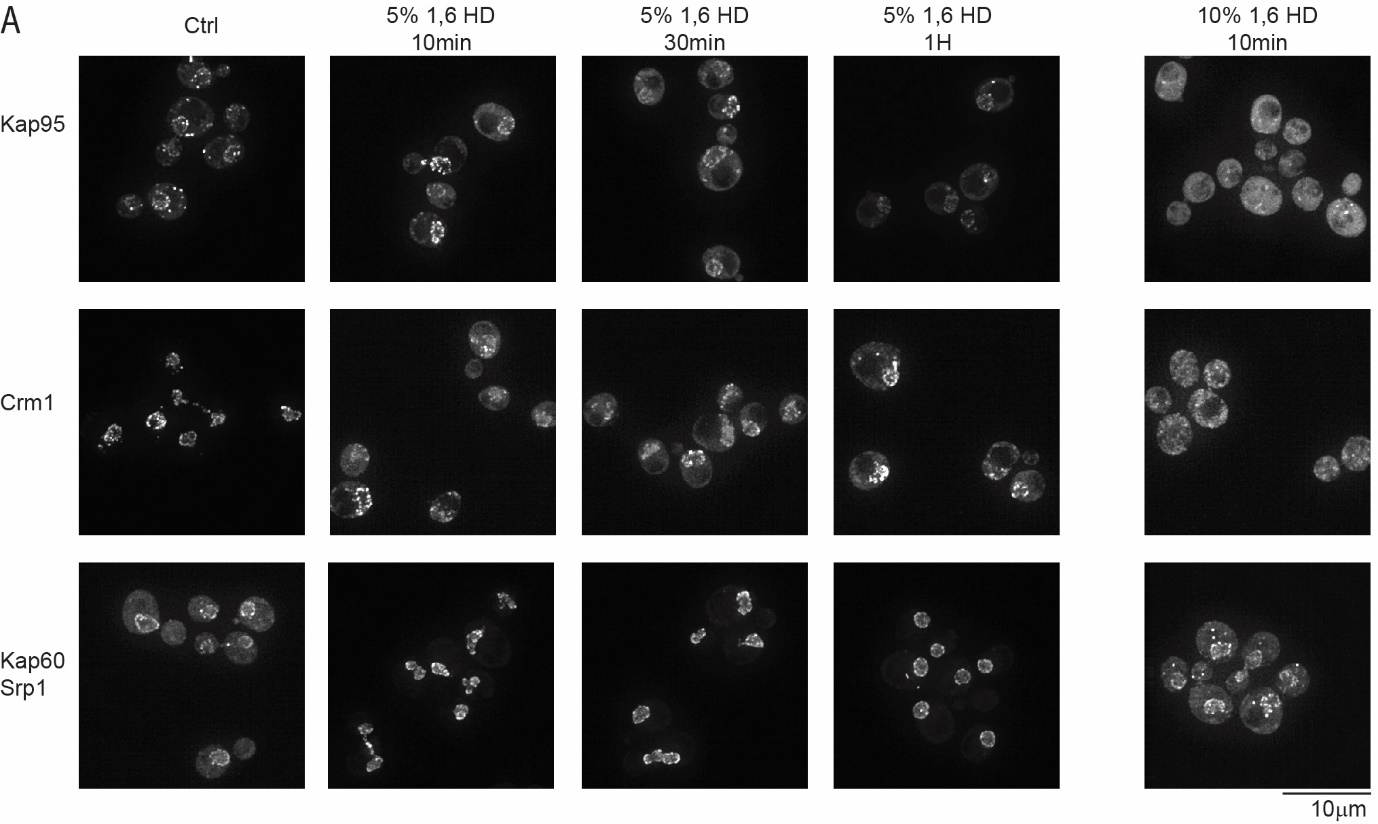


**Supplementary Figure 5.** Fluorescence images of endogenously GFP-tagged Kap95, Kap60 and Crm1 after exposure to 1,6HD as indicated on the top. Representative images of two independent replicates. The scale bar is 10μm

**
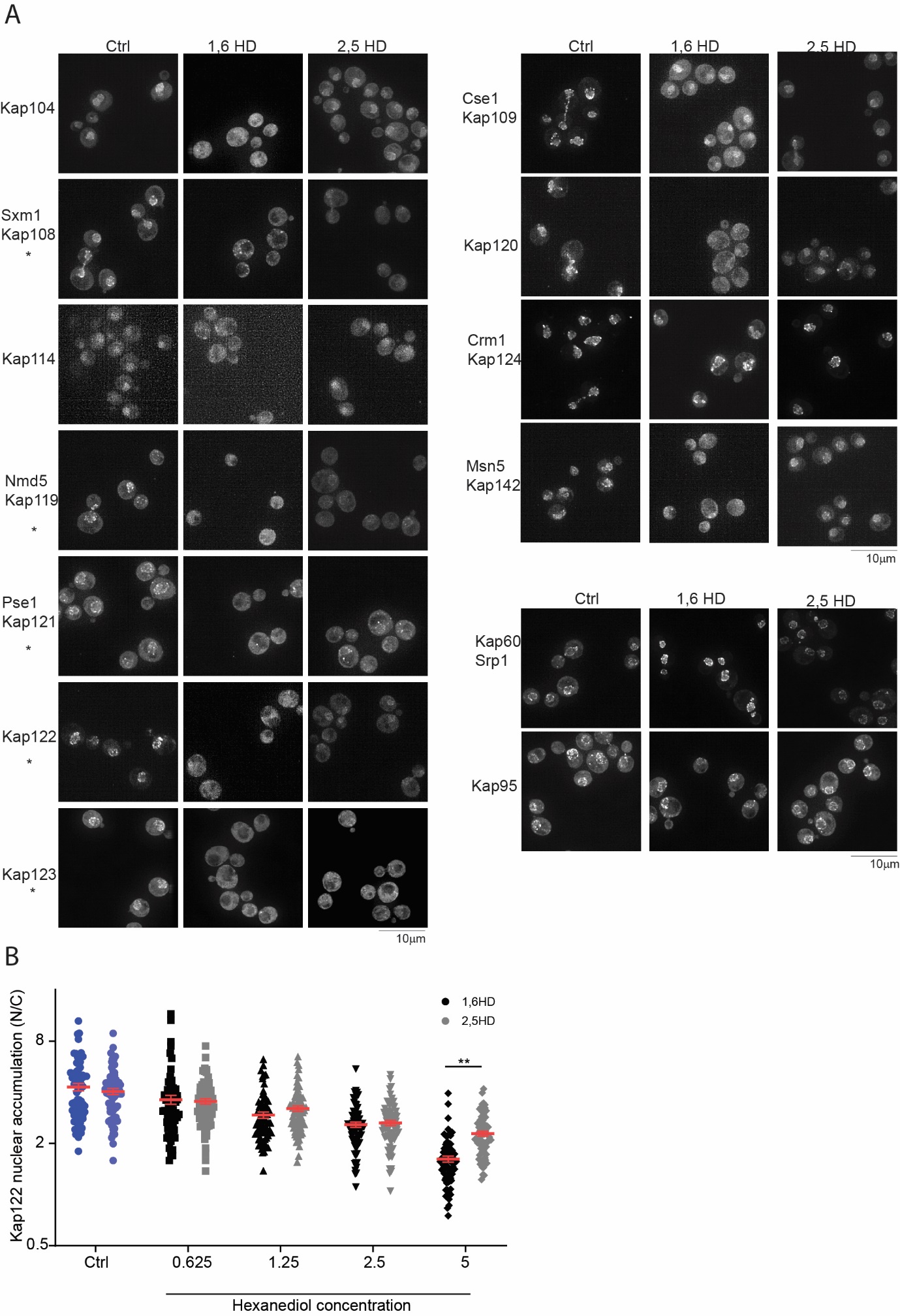
**

**Supplementary Figure 6.** (A) Fluorescence images of endogenously GFP-tagged NTRs after 10 min exposure with either 5% 1,6HD (middle, as I Fig 4A) or 5% 2,5HD (right). Representative images of three independent replicates. The scale bar is 10μm. (B) Nuclear accumulation of Kap122-GFP in yeast cells exposed for 10 min to the indicated concentrations of either 1,6HD (as in Fig 4B) or 2,5HD. Non-parametrical Kruskal-Wallis with Dunn’s multiple comparison test comparing treatment to control was used to calculate statistical significance. Mean and SEM of three independent experiments; 70 cells per condition were analysed; P-values from One-way ANOVA with Tukey’s multiple comparison test **<0,005.
